# Intracranial Human BT12 Glioblastoma Xenograft is [^18^F]FET PET Negative but 6‑[^18^F]Fluoronicotinic Acid PET Positive: Exploring a Novel Approach for Clinical Glioblastoma Imaging

**DOI:** 10.1021/acs.molpharmaceut.6c00279

**Published:** 2026-06-09

**Authors:** David Ekwe, Abiodun Ayo, Xiaoqing Zhuang, Pyry Dillemuth, Tomi T. Airenne, Emel Bakay, Petter Lövdahl, Jonne Kunnas, Lu Bai, Johan Rajander, Tiina A. Salminen, Jessica M. Rosenholm, Pirjo Laakkonen, Xiang-Guo Li

**Affiliations:** † Turku PET Centre, 8058 University of Turku, 20521 Turku, Finland; ‡ Translational Cancer Medicine Research Program, Facuty of Medicine, University of Helsinki, 00014 Helsinki, Finland; § Laboratory Animal Centre, HiLIFE, University of Helsinki, 00014 Helsinki, Finland; ∥ Department of Chemistry, University of Turku, 20520 Turku, Finland; ⊥ Structural Bioinformatics Laboratory and InFLAMES Research Flagship, Biochemistry, Faculty of Science and Engineering, 1040 Åbo Akademi University, 20520 Turku, Finland; ○ Pharmaceutical Sciences Laboratory, Department of Natural and Health Sciences, Faculty of Science and Engineering, Åbo Akademi University, 20520 Turku, Finland; ◧ Accelerator Laboratory, Åbo Akademi University, 20500 Turku, Finland; ◪ iCAN Flagship, University of Helsinki, 00014 Helsinki, Finland; ⬕ Turku PET Centre, Turku University Hospital, 20520 Turku, Finland; □ InFLAMES Research Flagship, University of Turku, 20520 Turku, Finland

**Keywords:** fluorine-18, 6-[^18^F]fluoronicotinic acid, [^18^F]FET, glioblastoma, monocarboxylate transporters, positron emission tomography

## Abstract

Glioblastoma is the most aggressive primary brain tumor in adults with a median survival of less than 2 years. Radiolabeled amino acids like *O*-(2-[^18^F]­fluoroethyl)-*L*-tyrosine ([^18^F]­FET) have tremendous value in the diagnosis and treatment monitoring of gliomas with positron emission tomography (PET). However, the existence of [^18^F]­FET PET negativity in 10–30% of patients with glioma underscores the need for alternative radiotracers. We recently reported that 6-[^18^F]­fluoronicotinic acid ([^18^F]­FNA) PET can clearly delineate intracranial human glioblastoma xenograft in a mouse model and that monocarboxylate transporters 1 and 2 (MCT1/2) mediate tumor uptake. This study evaluated the potential clinical utility of [^18^F]­FNA by comparing its PET imaging performance with the current clinical amino acid radiotracers, [^18^F]­FET and [^11^C]­methionine ([^11^C]­MET), as benchmarks. Orthotopic human glioblastoma xenograft models were prepared in mice for radiotracer comparisons and MCT blocking studies. Dynamic PET imaging, ex vivo biodistribution, brain tissue autoradiography, and histological staining were performed. In silico docking was also performed to analyze the interactions between MCT1 and FNA. The tumor was distinctly visualized on PET with [^18^F]­FNA, whereas [^18^F]­FET did not provide discernible tumor images in the same mice. Despite the negative [^18^F]­FET PET results, the tumor was [^11^C]­MET PET positive. In vivo blocking experiments indicated that MCT1, MCT2, and MCT4 are important transporters but may contribute in unequal measures to [^18^F]­FNA tissue uptake. In vivo blocking with AZD3965, a dual inhibitor of MCT1 and MCT2, reduced [^18^F]­FNA tumor uptake by 68–77%, while blocking MCT4 reduced uptake by 23%. In silico analysis supports ligand–protein interaction between FNA and MCT1. We were not able to visualize the human glioblastoma xenografts in mice by [^18^F]­FET PET, but tumor uptake was detected with [^18^F]­FNA and [^11^C]­MET PET. MCT1, MCT2, and MCT4 have significant roles in [^18^F]­FNA uptake in glioblastoma and other tissues. [^18^F]­FNA could be an alternative approach for clinical PET imaging of glioblastoma based on a biological mechanism completely different from current clinical approaches.

## Graphical abstract

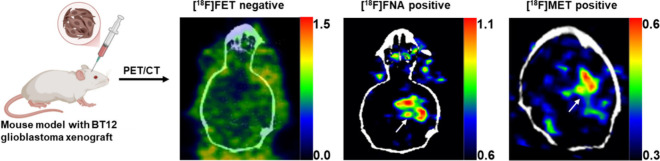

## 1 Introduction

Positron emission tomography (PET) is a useful modality for in vivo molecular profiling and treatment-response follow-up of brain tumors.^1–3^ PET can differentiate brain tumor recurrence from treatment-induced changes,^4^ which is a challenge in magnetic resonance imaging. Currently, amino acid–based radiopharmaceuticals, including [^11^C]­methionine ([^11^C]­MET) and *O*-(2-[^18^F]­fluoroethyl)-*L*-tyrosine ([^18^F]­FET) (Figure 1), are the main options for clinical PET imaging of brain tumors, as indicated by the guidelines from the PET task force of the Response Assessment in Neurooncology Working Group (PET/RANO), the European Association of Nuclear Medicine (EANM), the Society of Nuclear Medicine and Molecular Imaging (SNMMI), and the European Association of Neuro-Oncology (EANO).^5,6^ [^18^F]­FET PET has demonstrated tremendous value in several aspects of glioma management, including tumor delineation for radiotherapy planning, and early response assessment to chemotherapy.^1^ However, enhanced [^18^F]­FET uptake has not been observed in approximately 30% of low-grade gliomas and 5% of high-grade gliomas at primary diagnosis;^7^ in some other cohorts, approximately 10% of gliomas were [^18^F]­FET negative.^8^ Moreover, [^11^C]­MET availability is restricted to facilities with on-site cyclotrons. These limitations highlight the need for alternative approaches for glioma PET imaging. Our strategy is to develop ^18^F-labeled PET tracers transported by monocarboxylate transporters (MCTs) for neuro-oncological PET imaging. MCTs play crucial roles in disease and health,^9^ and MCT1 is the dominant subtype of MCTs expressed in the blood–brain barrier. As reported, MCT1 and MCT4 are overexpressed in some glioblastomas,^10^ and MCT2 expression has been observed in the blood–brain barrier, astrocytes, and neurons. Thus, there may be opportunities for developing radiotheranostics for brain tumor care via MCT-mediated uptake mechanisms. In this context, we recently found that 6-[^18^F]­fluoronicotinic acid ([^18^F]­FNA) PET can clearly visualize and delineate patient-derived BT12 glioblastoma xenograft in a mouse model, and MCT1/2 play important roles in tumor uptake.^11^ [^18^F]­FNA is straightforward to produce, stable in blood circulation, and has a favorable biodistribution pattern. However, extensive preclinical evaluations are still needed to support clinical translation. In this work, we report that [^18^F]­FET PET cannot visualize intracranial BT12 glioblastoma xenograft in the mouse model, while [^18^F]­FNA PET can. Despite [^18^F]­FET PET negativity, another amino acid tracer, [^11^C]­MET PET, was positive in the same tumor model. Furthermore, we investigated the involvement of MCT1, MCT2, and MCT4 in [^18^F]­FNA tumor uptake by preadministration of the corresponding inhibitors AZD3965 for MCT1/2 and AZD0095 for MCT4 in mice. AZD3965 is a potent inhibitor for MCT1 and exhibits 6-fold selectivity toward MCT2.^12^ AZD0095 is a MCT4 subtype selective inhibitor and has >1000-fold selectivity toward MCT1.^13^ [^18^F]­Fluorobenzoic acid ([^18^F]­FB) (Figure 1), a compound with a chemical structure similar to that of [^18^F]­FNA but lacking a nitrogen atom, was also studied to clarify the structural requirement for the nitrogen atom of [^18^F]­FNA for MCT transport. The interactions between MCT1 and FNA (the nonradioactive counterpart of [^18^F]­FNA) were further demonstrated by in silico docking analysis.

**Figure 1:**
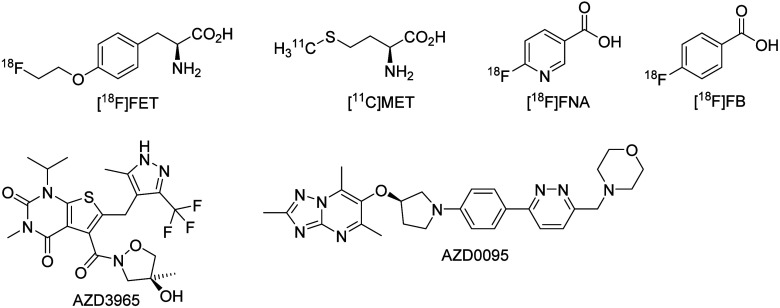
Chemical structures of [^18^F]­FET, [^11^C]­MET, [^18^F]­FNA, [^18^F]­FB, AZD3965, and AZD0095.

## 2 Experimental Section

### 2.1 Materials and General Methods

The precursor molecule *N*,*N*,*N*-trimethyl-5-((4-nitrophenoxy)­carbonyl)-pyridin-2-aminium trifluoromethanesulfonate for [^18^F]­FNA radiosynthesis was custom-synthesized by R & S Chemicals (Kannapolis, NC, USA). [^18^F]­FET, and [^18^F]­FB were prepared as previously reported.^14,15^ The precursor compound (2*S*)-O-(2′-tosyloxyethyl)-*N*-trityl-tyrosine-*tert*-butyl ester (TET) for [^18^F]­FET, *tert*-butyl 4-*N*,*N*,*N*-trimethylammoniumbenzoate triflate for [^18^F]­FB radiosynthesis, and reference compounds *L*- and *D*-FET were purchased from ABX GmbH, Radeberg, Germany. The other chemicals and reagents were obtained from Merck (Darmstadt, Germany). Clinical grade [^11^C]­MET was produced at the Turku PET Centre, University of Turku, Finland. This study used a mouse model with intracranial glioblastoma xenograft derived from human BT12 tumor cells,^16^ adhering to protocols approved by the Finnish National Project Authorisation Board with permission numbers ESAVI/10262/2022 and ESAVI/3630/2023.

### 2.2 Radiosynthesis of [^18^F]­FNA

[^18^F]­FNA was prepared as previously reported.^11^ [^18^F]­fluoride was produced in-house, and was captured on a Sep-Pak Accell Plus QMA Plus Light anion-exchange cartridge (130 mg, Waters, Milford, MA, USA), which had been preconditioned with 0.5 M aqueous potassium carbonate (2.0 mL) and water (5.0 mL) (TraceSELECT, Honeywell, Charlotte, NC, USA). The elution of [^18^F]­fluoride was carried out using Kryptofix 2.2.2 (9.2 mg, 23.1 μmol) and potassium carbonate (1.6 mg, 15.4 μmol) in Milli-Q water (76.9 μL) and acetonitrile (1.9 mL). The eluate was then dried under nitrogen at 120 °C, after which precursor *N*,*N*,*N*-trimethyl-5-((4-nitrophenoxy)­carbonyl)-pyridin-2-aminium trifluoromethanesulfonate (10.0 mg, 22.2 μmol) in 0.8 mL acetonitrile was added. The mixture was kept at 37 °C for 10 min and then diluted with water (1.0 mL). The intermediate product, 6-[^18^F]­fluoronicotinic acid 4-nitrophenyl ester, was purified using high performance liquid chromatography (HPLC, PU-4180, JASCO Corporation, Tokyo, Japan) equipped with a radioactivity detector (DM Automation, Nykvarn, Sweden) and a reversed-phase C18 column (Jupiter Proteo, 250 × 10 mm, 5 μm, 90 Å; Phenomenex, Torrance, CA, USA) at a flow rate of 4 mL/min. Solvent A consisted of 0.1% trifluoroacetic acid (TFA) in water, while solvent B was 0.1% TFA in acetonitrile. The HPLC gradient ranged from 45 to 70% B over 0–13 min. The HPLC fraction containing 6-[^18^F]­fluoronicotinic acid 4-nitrophenyl ester was diluted with 30 mL of water and extracted onto an Oasis HLB Plus Light cartridge (Waters, Milford, MA, USA). The ester was hydrolyzed on the cartridge by passing 1.0 M NaOH (580.0 μL) through the HLB cartridge, which was left for 10 min before [^18^F]­FNA was eluted with 2.0 mL of water into a vial containing 2.0 M HCl (140.0 μL) and 2.0 M phosphoric acid (125.0 μL). Postsynthesis, the identity and radiochemical purity of [^18^F]­FNA were verified using analytical HPLC (LC-40D, Shimadzu Corporation, Kyoto, Japan). Accordingly, a sample of 0.5–0.8 MBq of [^18^F]­FNA was injected into a C18 reversed-phase column (Jupiter Proteo, 250 × 4.6 mm, 5 μm, 90 Å; Phenomenex). The results were compared with cold reference HPLC results, where 20–50 nmol of commercial 6-fluoronicotinic acid (Merck, Rahway, NJ, USA) in water was used. Solvent A was 0.1% TFA in water, and solvent B was 0.1% TFA in acetonitrile. The HPLC gradient started at 10% B and increased to 50% B over 0–10 min, at a flow rate of 1.5 mL/min, monitored by radioactivity and ultraviolet (UV) detection at 220 and 254 nm.

### 2.3 Radiosynthesis of [^18^F]­FET

[^18^F]­FET was prepared according to a previously published procedure.^14^ An aqueous [^18^F]­fluoride solution was loaded on a preconditioned Sep-Pak Accell Plus QMA Plus light cartridge (130 mg, Waters, WAT023525). The cartridge was preconditioned by sequential washing with 10 mL of 0.5 M Na_2_HCO_3_ and 10 mL of water. [^18^F]­Fluoride was eluted into a reaction vial using 1.2 mL of a K_2_CO_3_/K_222_ solution (14.18 mg/3.27 mg) in CH_3_CN/H_2_O (10:1 by volume). The solvent was removed via azeotropic drying with CH_3_CN at 115 °C. Subsequently, 4 mg of the precursor, (2*S*)-O-(2′-tosyloxyethyl)-*N*-trityl-tyrosine-*tert*-butyl ester (TET), dissolved in 1 mL CH_3_CN, was added to the reaction vial and heated at 90 °C for 10 min. After cooling to 50 °C, 1 mL of 2 M HCl was added, and the mixture was heated at 100 °C for 10 min. The reaction mixture was neutralized with NaOH solution and purified using a semipreparative HPLC column Jupiter 4 μm Proteo 90 Å (250 × 10 mm, Phenomenex, Torrance, CA, USA). The mobile phase consisted of 10% ethanol and 0.1% acetic acid in water and the flow rate was 4 mL/min. [^18^F]­FET was isolated and formulated in phosphate-buffered saline (PBS). Radiochemical purity was determined via analytical HPLC using a Jupiter Proteo column (4 μm, 90 Å, 250 × 4.6 mm). The column was eluted using a gradient protocol with mobile phases of solvent A (water containing 0.1% TFA) and solvent B (0.1% TFA in CH_3_CN). The gradient was programed from 15% to 50% solvent B over 12 min at a flow rate of 1 mL/min, with radioactivity and UV detection at 220 nm.

### 2.4 Radiosynthesis of [^18^F]­FB

[^18^F]­FB was prepared according to a published method with slight modifications.^15^ The complex of [^18^F]­fluoride with K_2_CO_3_/K_222_ was prepared and dried similarly as described above. Precursor *tert*-butyl 4-*N*,*N*,*N*-trimethylammoniumbenzoate triflate (5.00 mg in 1 mL of CH_3_CN) was added and the mixture was heated at 90 °C for 15 min. After cooling to 50 °C, the solution was diluted with 0.8 mL of 72 mM HCl and 0.3 mL of CH_3_CN and purified by semipreparative HPLC using a Jupiter 4 μm Proteo 90 Å column (250 × 10 mm, Phenomenex, Torrance, CA, USA) with 70% CH_3_CN (containing 0.1% TFA) in water (containing 0.1% TFA) at a flow rate of 4 mL/min. The ^18^F-labeled intermediate *tert*-butyl 4-[^18^F]­fluorobenzoate was collected in 25 mL of water and trapped in a preconditioned Oasis HLB cartridge (30 mg, preconditioned with 10 mL of ethanol and 10 mL of water). After washing with 5 mL of water, the intermediate was eluted into a separate reaction vial using 0.5 mL of ethanol. Hydrolysis was performed by adding 0.5 mL of 1 M HCl and heated at 100 °C for 5 min to yield the final product [^18^F]­FB. The solution was neutralized with 0.6 mL of 1 M NaOH. The final product was formulated in PBS, and the content of ethanol was 9% at maximum to ensure that the final product formulation was suitable for animal administration. Radiochemical purity was determined by analytical HPLC with a Jupiter Proteo column (4 μm, 90 Å, 250 × 4.6 mm) using a gradient elution with water (containing 0.1% TFA, solvent A) and CH_3_CN (containing 0.1% TFA, solvent B), 40–80% solvent B over 10 min at a flow rate of 1.5 mL/min, with radioactivity and UV detection at 254 nm.

### 2.5 Glioblastoma Mouse Model Preparation and Animal PET Imaging

Mouse model with intracranial BT12 glioblastoma xenogragfts was prepared according to a published method.^11^ Briefly, 4–5 weeks old female immunocompromised mice (Rj:NMRI-Foxn1nu/nu, Janvier Laboratory, Le Genest-Saint-Isle, France) were intracranially inoculated with human-derived glioblastoma BT12 cells (200,000 cells in 5 μL of 0.9% saline) using a 10-μL Hamilton syringe (Hamilton Company, Reno, NV, USA) mounted on a stereotactic frame (World Precision Instruments, Sarasota, FL, USA). The cells were implanted on the right hemisphere at coordinates approximately 2 mm posterior and 2.0 mm lateral to the bregma, and at a depth 2.5 mm below the dura mater. For the duration of the procedure, the mice were maintained under 2% isoflurane and kept warm with a heating pad set at 37 °C. PET imaging was conducted at day 21 after tumor cell inoculation. The study design is outlined in Supplemental Figure S1. Molecubes small-animal PET and CT systems (Molecubes NV, Gent, Belgium) were employed for imaging. As previously reported,^11^ the PET/CT imaging protocols began with a 10 min high-resolution CT scan, followed by intravenous radiotracer injection via tail vein cannulation. CT was performed prior to PET to ensure immediate tissue collection with less radioactivity in the tissue samples. CT and PET were acquired sequentially using the same imaging bed without repositioning the animals. The injected doses were 4.48 ± 0.41 MBq (n = 13) for [^18^F]­FNA, 4.46 ± 0.41 MBq (n = 4) for [^18^F]­FET, 4.14 ± 0.53 MBq (n = 3) for [^18^F]­FB, and 3.45 ± 0.55 MBq (n = 4) for [^11^C]­MET. Dynamic PET data were collected for 60 min in list mode, imaging two mice simultaneously. For in vivo blocking experiments, the procedure remained consistent, with the addition of MCT1/2 dual inhibitor AZD3965 (1.1 mg/kg bodyweight) or MCT4 inhibitor AZD0095 (1.1 mg/kg), administered intravenously 15 min prior to [^18^F]­FNA injection. During the whole PET/CT imaging procedure, mice were kept under anesthesia with a continuous inhalation of 1–2% isoflurane and heating. PET/CT data were analyzed in the same way as previously reported,^11^ and our in-house–developed Carimas 2.10 software (Turku PET Centre, Finland, www.turkupetcentre.fi/carimas/) was used as the major analysis tool. The details for PET image analysis were described in the Supporting Information section PET/CT Image Analysis.

### 2.6 Ex Vivo Biodistribution and Brain Tissue Autoradiography

Following PET imaging, the mice were deeply anesthetized with isoflurane inhalation and euthanized via cardiac puncture through the left ventricle, followed by cervical dislocation. The animals were then perfused with 10 mL of PBS through the left ventricle to eliminate residual blood radioactivity. Tissue samples were collected, and their radioactivity was measured using a Wizard gamma counter (Wizard^2^ 2480, PerkinElmer, Waltham, MA, USA). The radioactivity measurements were normalized based on the injected dose per animal weight, tissue sample weight, and radioactivity decay. The injected dose was adjusted for any remaining radioactivity in the tail and cannula. Results are presented as the percentage of injected radioactivity dose per gram (%ID/g) of tissue. Mouse brains were snap-frozen in dry ice-cooled isopentane and sectioned into 20-μm thick cryosections. The 20-μm sections were thaw-mounted on microscopy slides and exposed overnight to a phosphor imaging plate (BAS-TR2025, Fujifilm, Tokyo, Japan) within lead shielding. Digital autoradiographs were obtained using a BAS-5000 scanner (Fujifilm, Tokyo, Japan) and analyzed using Carimas. Results were expressed as photostimulated luminescence per square millimeter (PSL/mm^2^) with background correction applied. Following autoradiography, the same tissue sections underwent standard H&E staining at the Histology Core Facility at the University of Turku.

### 2.7 In Vivo Stability and Blood Radioactivity Analysis

After PET imaging (60 min after radiotracer injection), mouse blood was collected in heparinized tubes. Plasma was separated from the blood cells by centrifugation (2,100 × *g*, 5 min). Acetonitrile-induced protein precipitation and subsequent centrifugation (14,000 × *g*, 3 min, room temperature) yielded protein-free plasma. A Wizard gamma counter was used to measure radioactivity in the isolated blood components. HPLC analysis with a radioactivity detector was performed on the protein-free plasma supernatant. The analysis employed a reversed-phase C18 column (Phenomenex Jupiter Proteo, 250 × 10 mm, 5 μm, 90 Å) with a flow rate of 5 mL/min. Solvents comprised 0.1% TFA in water (solvent A) and 0.1% TFA in acetonitrile (solvent B). For [^18^F]­FET, the following method was used: a 15 min elution gradient from 15% to 35% solvent B, starting 1 min after injection, followed by a 2 min gradient to 80% solvent B, whose concentration was maintained for 2 min. For [^18^F]­FB, a 12 min elution gradient from 40% to 70% solvent B was used and urine was analyzed by diluting the sample with water.

### 2.8 In Silico Docking Analysis of FNA and MCT1

The programs from the Schrödinger package were used for docking. The MCT1 structure was prepared for docking using the Protein Preparation Workflow, and the default parameters were used with the following exceptions: 1) delete waters beyond hets 4.00 Å, 2) minimize hydrogens only, and 3) delete waters with fewer than three bonds to nonwaters. The ligands were prepared using the LigPrep tool and default parameters were used with the following exceptions: 1) no desalt and 2) stereoisomers: generate at most two per ligand. The OPLS4 (Optimized Potentials for Liquid Simulations 4) force field was used. For docking with Glide, the receptor grid for MCT1 was created using the Receptor Grid Generation tool, and the docking site was chosen by selecting the lactate ligand (PDB code 6LZ0, chain A) and the “Dock ligands with similar size” option. Docking was performed using the default Glide parameters (maximum 10 poses were written out). Because the mouse MCT1 (mMCT1) and rat MCT1 (rMCT1) models were highly similar to the cryoEM structure of human MCT1 (hMCT1; PDB code 6LZ0), and all the residues in close proximity to the cocrystallized lactate ligand were conserved in mMCT1, rMCT1, and hMCT1, FNA was docked only to the hMCT1 structure. All figures related to the structural representation of the docking results were prepared using the PyMOL Molecular Graphics System (Version 3.0 Schrödinger, LLC) and Inkscape (https://inkscape.org).

### 2.9 Statistical Analysis

We used GraphPad Prism version 10.4.0 (GraphPad Software, Boston, USA) for statistical analyses. Results are presented as mean ± standard deviation. Paired *t* test was to assess the differences when comparing tracer uptake between tissues in the same animal, while differences between independent data sets were assessed using the unpaired Student’s *t* test, with P values <0.05 considered statistically significant.

## 3 Results

### 3.1 Radiopharmaceutical Chemistry

[^18^F]­FNA was synthesized with a decay-corrected radiochemical yield of 29.2 ± 7.3% (n = 5). As examined by analytical HPLC, the radiochemical purity was 99.4 ± 0.5% (n = 5) and the molar activity was 144.9 ± 48.5% (n = 4). The average synthesis time was 104.4 ± 11.3 min (n = 5). [^18^F]­FET radiosynthesis involved nucleophilic substitution with [^18^F]­fluoride, followed by acid hydrolysis with HCl, and purification by HPLC. Starting with 3.5–5.5 GBq of [^18^F]­fluoride, [^18^F]­FET was obtained with a decay-corrected radiochemical yield of 47.87 ± 10.46% (n = 3), with radiochemical purity >99%. Four hours after radiosynthesis, the radiochemical purity remained above 99%. The total synthesis time was 99 ± 7 min (n = 3). The molar activity was 35.67 GBq/μmol at the end of synthesis. In addition, the (*S*)-enantiomer (corresponding to the *L*-configuration) of [^18^F]­FET was confirmed (Figure 2). The radiosynthesis of [^18^F]­FB yielded decay-corrected radiochemical yields of 38.38% and 30.12% from the two batches. The radiochemical purity was 97.39 ± 1.19% within a total synthesis time of 114 ± 3 min. After 6 h, the radiochemical purity remained high at 97.97 ± 0.87% (Supplemental Figure S2).

**Figure 2:**
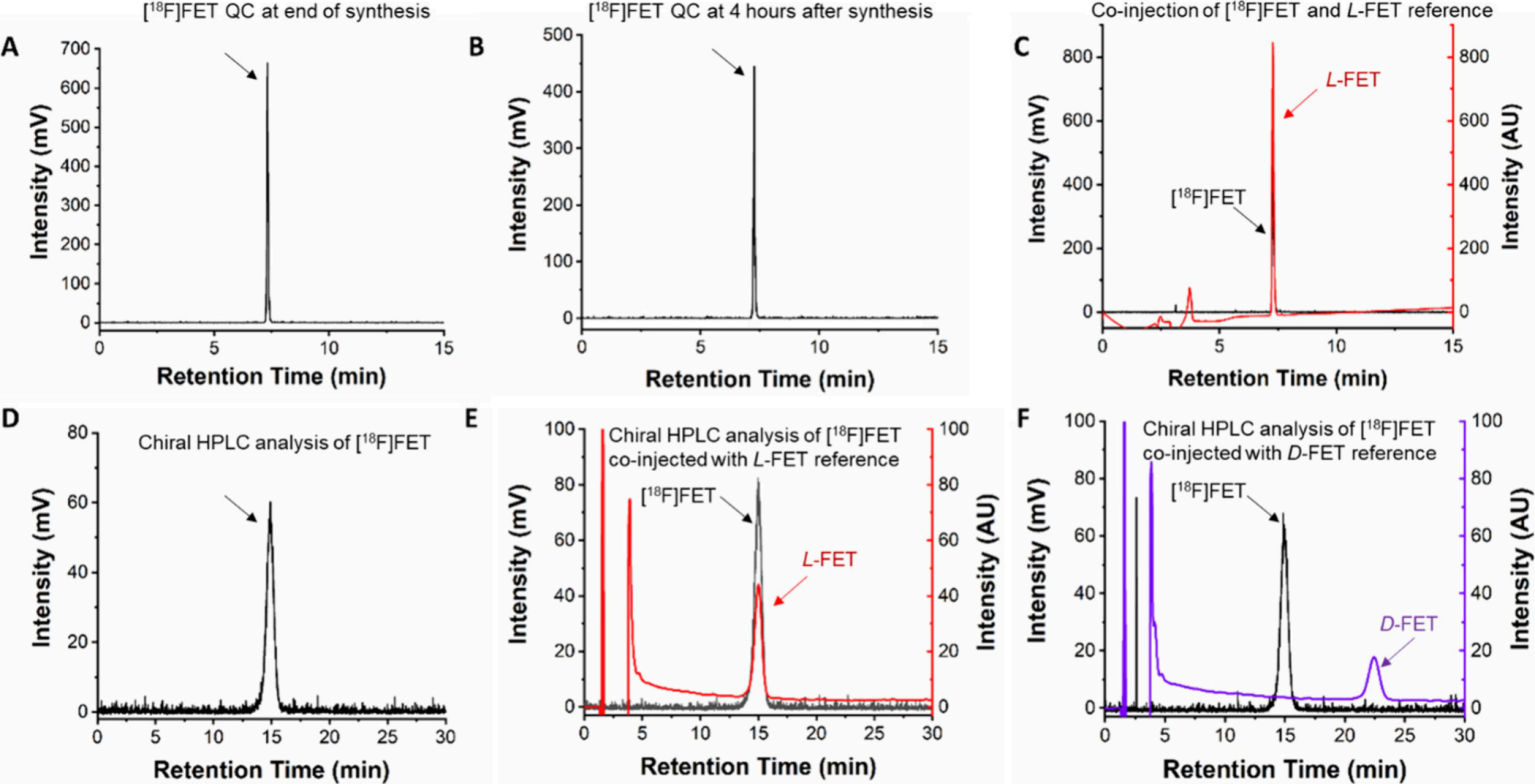
Analytical HPLC chromatograms of [^18^F]­FET and the nonradioactive reference FET. (A) Radiochemical purity measurement of [^18^F]­FET with HPLC at the end of synthesis as quality control (QC). (B) Radiochemical purity measurement of [^18^F]­FET with HPLC at 4 h after synthesis. (C) HPLC analysis of the end product [^18^F]­FET spiked with the reference compound (2*S*)-*O*-(2′-fluoroethyl)-*L*-tyrosine (*L*-FET) to confirm the chemical identity of [^18^F]­FET using both radioactivity and UV detection. (D) [^18^F]­FET enantiopurity was >99% as measured by analytical chiral HPLC under radioactivity detection. (E) Chiral HPLC analysis of [^18^F]­FET coinjected with the reference compound *L*-FET to confirm the chemical identity and (*S*)-enantiomer of [^18^F]­FET using both radioactivity and UV detection. (F) Chiral HPLC analysis of [^18^F]­FET co-injected with the reference compound (2*R*)-*O*-(2′-fluoroethyl)-*D*-tyrosine (*D*-FET) as a control experiment under both radioactivity and UV detection.

### 3.2 BT12 Glioblastoma Xenografts are [^18^F]­FET Negative but [^18^F]­FNA Positive

Animal study design was shown in Supplemental Figure S1. We performed a head-on-head comparison study between [^18^F]­FNA and [^18^F]­FET in the same mice (n = 3) and one additional mouse for each tracer. PET/CT imaging of [^18^F]­FNA in mice with BT12 intracranial glioblastoma xenograft showed that the tracer accumulated in the tumor tissue, providing a clear visualization of the tumor in all subjects (Figure 3A). The mean standardized uptake value was significantly higher in the tumor than in the contralateral healthy brain region (SUV_mean_ = 0.53 ± 0.15 vs 0.40 ± 0.09, respectively, P = 0.024). Similarly, the maximum standardized uptake value was significantly higher in the tumor than in the healthy brain (SUV_max_ = 0.83 ± 0.21 vs 0.58 ± 0.06, respectively, P = 0.023), yielding a tumor-to-brain ratio (TBR) of 1.44 ± 0.39 at 5–10 min after injection. In contrast, the glioblastoma xenografts were not discernible in the PET imaging with [^18^F]­FET in the same mice at any time frame during the 60 min dynamic imaging protocol (Figure 3B). This was despite the comparable healthy brain region having a similar SUV_mean_ (0.40 ± 0.08, P = 0.486) and SUV_max_ (0.66 ± 0.09, P = 0.168) as [^18^F]­FNA. However, the BT12 glioblastoma xenograft were [^11^C]­MET PET positive in the control experiments (Figure 3C).

**Figure 3:**
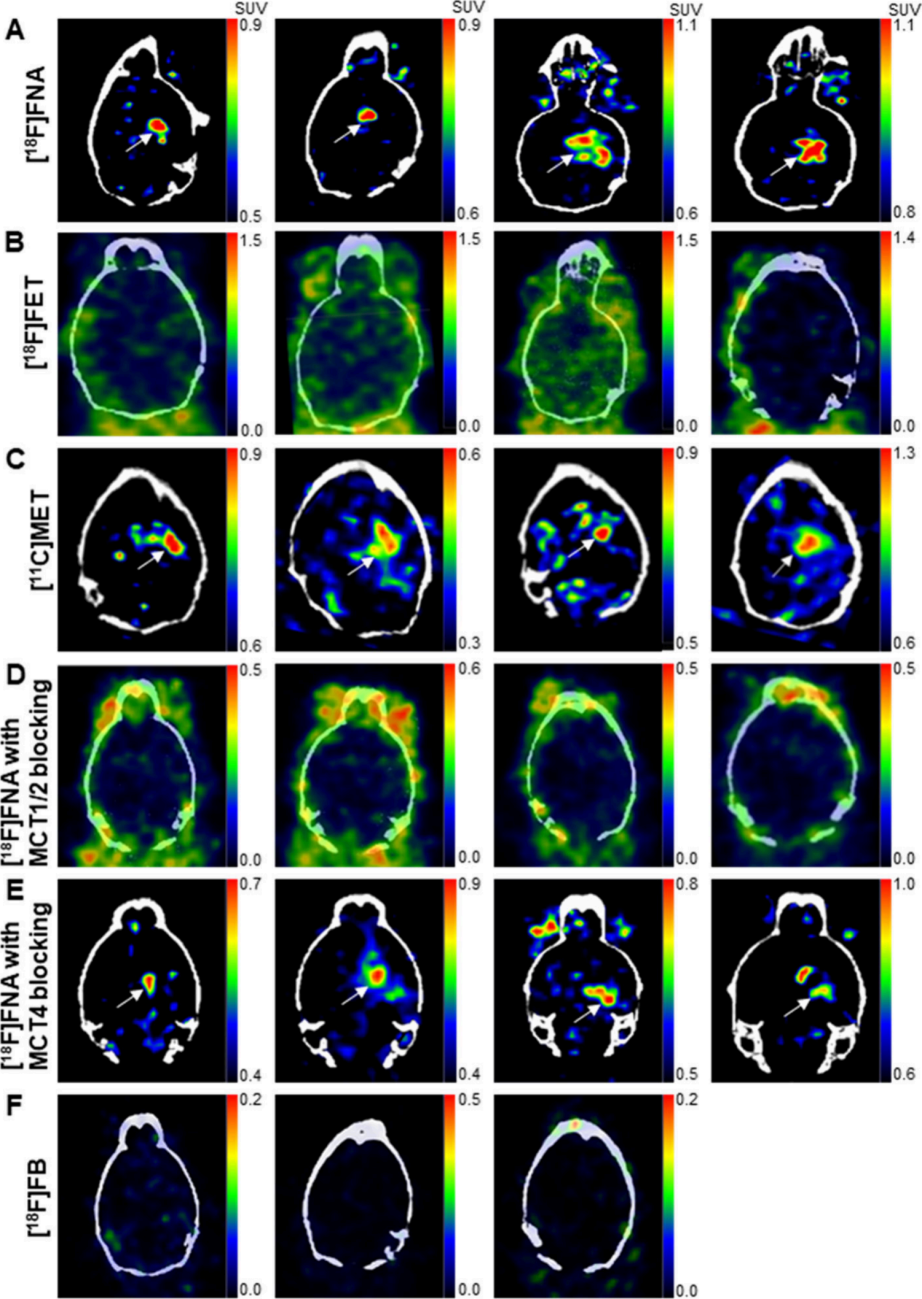
Time-weighted mean PET/CT images of intracranial glioblastoma xenograft in mice comparing the relative performance in tumor visualization with (A) [^18^F]­FNA during 4–30 min, (B) [^18^F]­FET during 20–60 min, (C) [^11^C]­MET during 4–30 min, (D) [^18^F]­FNA with MCT1/2 blocking during 4–30 min, (E) [^18^F]­FNA with MCT4 blocking during 4–30 min, and (F) [^18^F]­FB during 4–30 min. The brain tumor is indicated by white arrows. Each PET/CT image in each panel was from a different mouse.

Analysis of the time-activity curve (TAC) in the brain showed that [^18^F]­FNA rapidly accumulated in the tumor, which peaked approximately 10 min after injection, followed by a slow washout over time (Figure 4A). In the contralateral healthy brain region, tracer uptake followed a similar kinetic trajectory but remained consistently lower than in the tumor throughout the 60 min dynamic imaging. This is in line with the results we have observed previously.^11^ Interestingly, [^18^F]­FET showed a distinctively different kinetic profile (Figure 4B) compared with [^18^F]­FNA (Figure 4A). A consistent increase in the uptake and retention of [^18^F]­FET was observed in both the tumor and healthy brain regions throughout the imaging duration, with the tumor having a slightly higher tracer uptake (Figure 4B). However, the difference was not statistically significant, and their SUV curves remained closely aligned. [^18^F]­FNA tumor uptake compared with that of [^18^F]­FET showed that [^18^F]­FNA had a higher uptake at the early time points (Figure 4C). [^18^F]­FET had higher tumor uptake than [^18^F]­FNA starting from approximately 28 min postinjection, but the TBR remained similar as earlier time points, which did not allow tumor delineation. Furthermore, the uptake kinetics were clearly different in several organs and tissues except blood (Figures 5A–5F). In general, [^18^F]­FET uptake plateaued in the lungs, muscle, and liver after approximately 10 min after tracer injection, while [^18^F]­FNA showed a descending trend in those organs. Both tracers had quick clearance from the blood circulation (Figure 5D), and TBRs remained steady during 5–60 min of PET imaging in both cases (Figure 5G). The results of the ex vivo biodistribution revealed that [^18^F]­FET had significantly higher uptake in most of the examined tissues (Figure 6; Supplemental Table S1). In the bones, kidneys, and uterus, the uptake was not statistically different between the two groups. Notably, [^18^F]­FET uptake in the whole brain was significantly higher than [^18^F]­FNA (*P* < 0.019) 60 min after injection. The presence of glioblastoma xenografts was confirmed with hematoxylin and eosin (H&E) staining in all mice, and representative H&E and autoradiography images are shown in Supplemental Figure S3. In the autoradiography of mouse brain sections prepared 60 min after [^18^F]­FET injection, homogeneous radioactivity uptake in the whole section was observed, and the tumor tissue was not delineable either (Supplemental Figure S3A).

**Figure 4:**
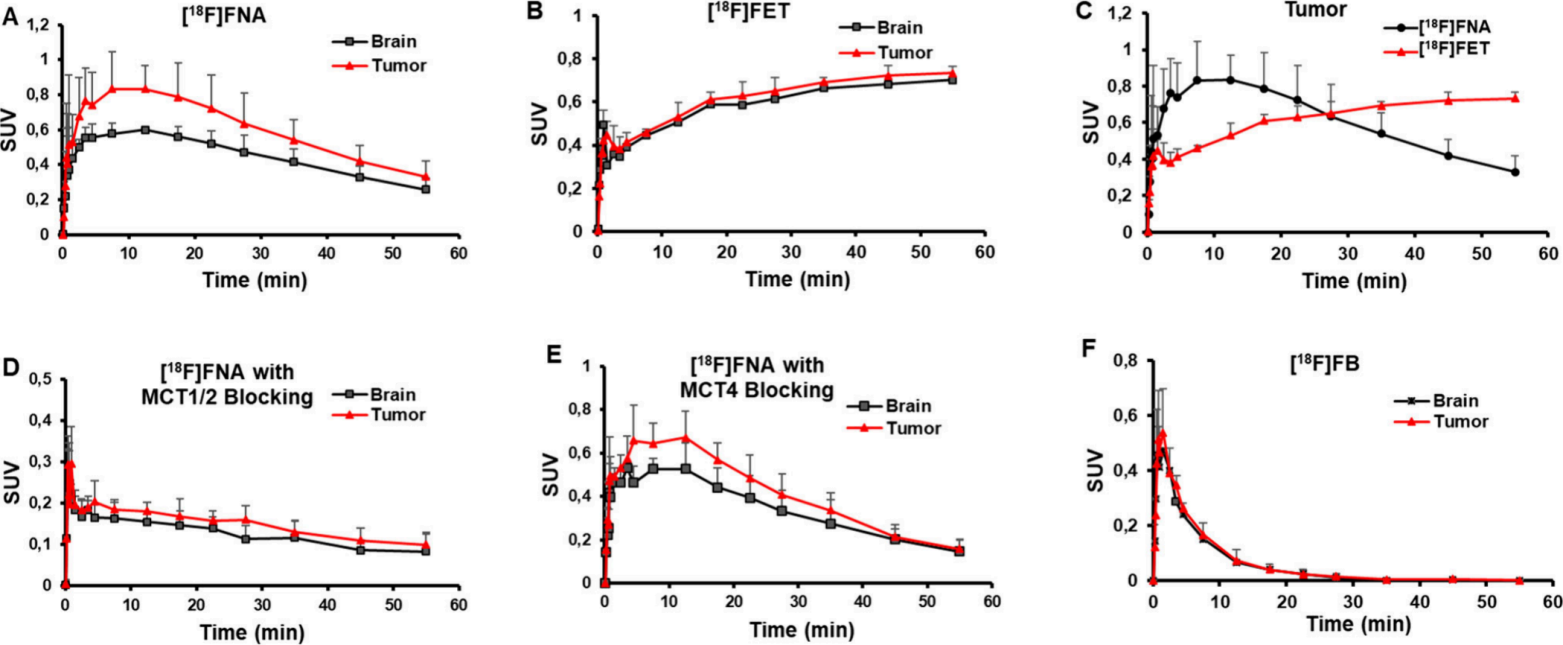
TACs from the brain and tumor of mice with glioblastoma showing the kinetics of different tracers. (A) [^18^F]­FNA. (B) [^18^F]­FET. (C) Tumor uptake of [^18^F]­FNA compared with that of [^18^F]­FET. (D) [^18^F]­FNA with MCT1/2 blocking. (E) [^18^F]­FNA with MCT4 blocking. (F) [^18^F]­FB.

**Figure 5:**
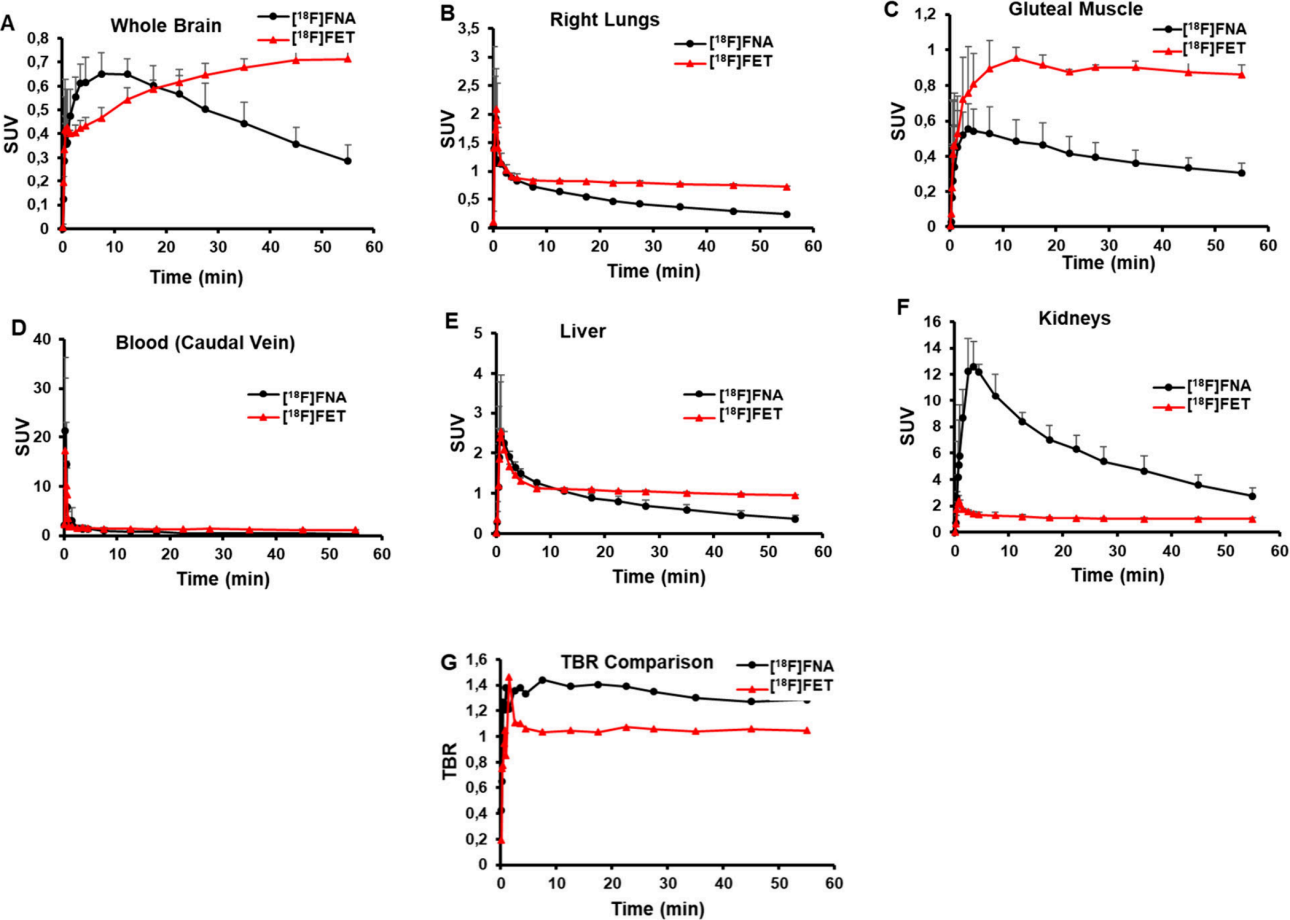
TACs of selected tissues (A–F) as measured with [^18^F]­FNA and [^18^F]­FET PET, and (G) TBR comparison between [^18^F]­FNA and [^18^F]­FET.

**Figure 6:**
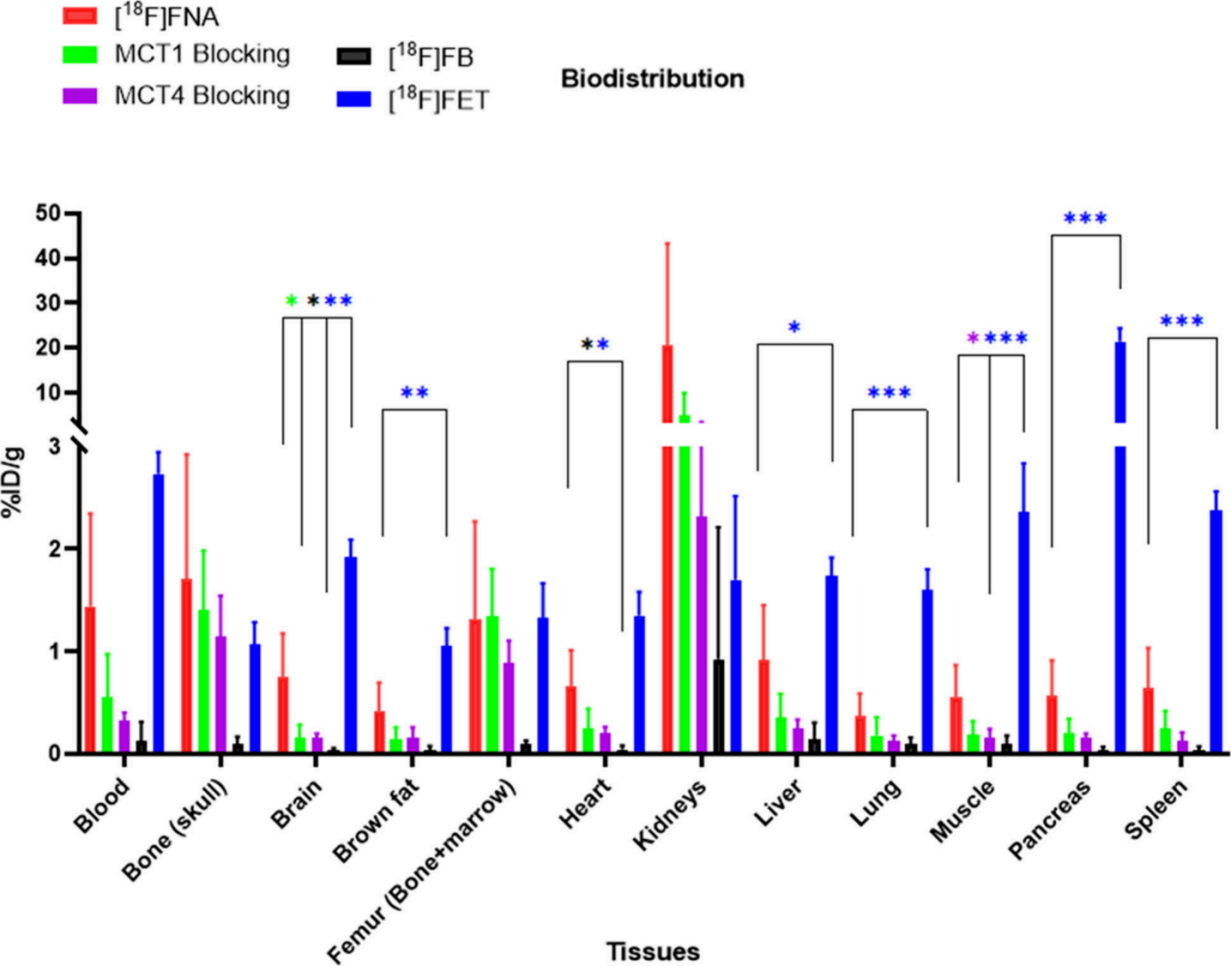
Histogram of ex vivo biodistribution of selected organs in mice with glioblastoma. Statistically significant differences between [^18^F]­FNA and other groups are indicated with color-coded asterisks according to group color (*P < 0.05, **P < 0.01, and ***P < 0.001). %ID/g denotes percentage of injected dose per gram of tissue weights.

### 3.3 MCT1/2 Blocking Reduces [^18^F]­FNA Uptake in Tumor and Other Tissues

Our previous data from four mice indicated that in vivo blocking using the MCT1/2 dual inhibitor AZD3965 significantly reduces [^18^F]­FNA uptake in glioblastoma xenografts.^11^ To confirm the previous finding, we performed similar blocking experiments in four other mice. In addition, TACs were analyzed not only for brain and glioblastoma but also for several other organs. In the blocking experiments with AZD3965 (1.1 mg/kg) administered 15 min before [^18^F]­FNA injection, the tumors could not be visualized on PET images (Figure 3D), and tumor tracer uptake was markedly reduced by 68–77% compared with the [^18^F]­FNA uptake under the nonblocking conditions (Figure 3A). This is evidenced by the significant decrease in the SUV_mean_ values in the tumor (0.17 ± 0.01, P = 0.017) and brain regions (0.16 ± 0.02, P = 0.009) as well as in the SUV_max_ values of the tumor (0.19 ± 0.02, P = 0.008) and brain (0.18 ± 0.04, *P* < 0.001) compared with their corresponding values in the nonblocked cohort. Consequently, the peak TBR decreased to 1.14 ± 0.31 at 5–10 min. The brain and glioblastoma uptake kinetics also showed a clear difference between the MCT1/2-blocked (Figure 4D) and nonblocked groups (Figure 4A). A statistically significant difference was observed in lungs and kidneys at each time point between 5–60 min postinjection, while the liver and muscle uptake remained unchanged (Figure 7). The cohort with MCT1/2 blocking exhibited significantly lower SUV in the brain, lungs, and kidneys (mean difference range: 0.19–0.47, 0.12–0.16, and 1.65–3.69, respectively; *P* < 0.05 across all time points) (Supplemental Table S2), according to the in vivo dynamic PET imaging data. However, in the lungs, the difference at two intermediate time frames, 20–25 and 25–30 min, did not reach statistical significance (P = 0.085 and 0.055, respectively).

**Figure 7:**
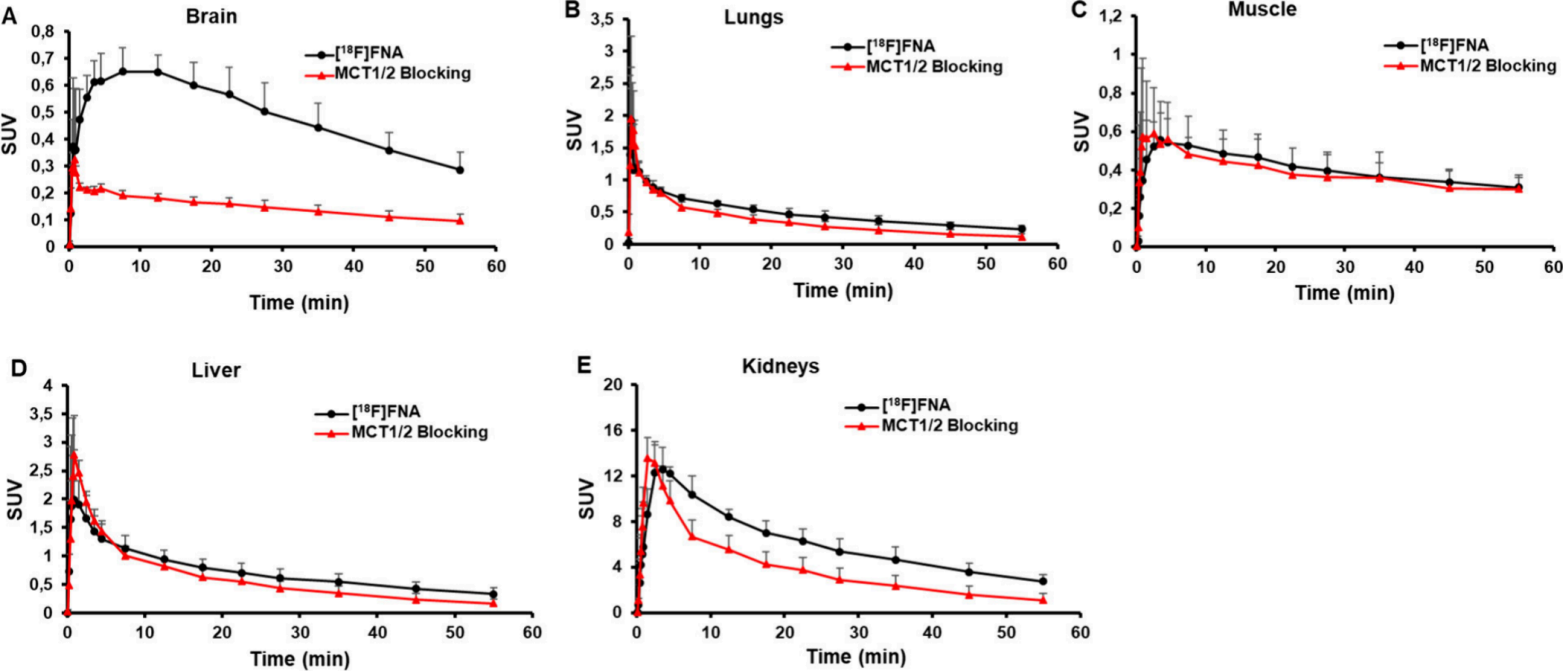
TACs of selected tissues comparing [^18^F]­FNA uptake in tissues with or without MCT1/2 blocking.

Next, we used in silico docking analysis to study the binding of FNA (the nonradioactive counterpart of [^18^F]­FNA) to the cryo-EM structure of human MCT1 (hMCT1) (Figure 8). In the published cryo-EM structure,^17^ FNA was predicted to bind to MCT1 in a position close to that of the bound lactate, which is the natural substrate of MCT1. Based on the analysis of the pose with the highest Emodel score, the key interactions include hydrogen bonds between the carboxylate group of FNA and the side chain of K38, S154, and R313 of MCT1, and the hydrogen bond between the nitrogen atom of FNA and the side chain of S371. Hydrophobic and van der Waals interactions seem to affect FNA binding as well, as FNA is within interaction distance (<4 Å) from the side chains of Y34, M151, F278, F367, and L374. Moreover, we docked nicotinic acid (the corresponding nonfluorinated natural compound) to MCT1, and the nicotinic acid pose with the highest Emodel score was in practice identical to the FNA pose when the fluorine atom was ignored (Data not shown).

**Figure 8:**
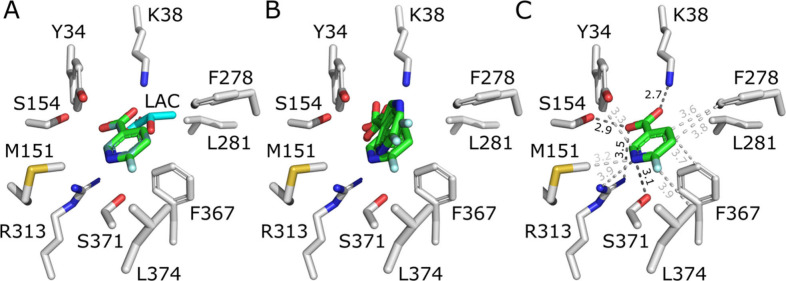
Predicted FNA binding to the “outward-open” conformation of hMCT1 (PDB code 6LZ0). The ligands (FNA, green carbon atoms; LAC (lactate), cyan carbon atoms) and key amino acid residues of the binding pocket (gray carbon atoms) are shown as sticks. (A) Binding comparison between LAC and FNA. (B) The three most likely FNA binding poses based on Emodel scores (−38.839, −37.973, and −37.434; more negative values represent tighter binders).^17^ (C) Key interactions of the most likely FNA pose (Emodel = −38.839; GlideScore = −6.333). Potential hydrogen bonds are indicated with gray dashes and weak interactions, including van der Waals and hydrophobic interactions, with light gray dashes. The distances are shown in angstroms.

### 3.4 In Vivo MCT4 Blocking Reduces [^18^F]­FNA Uptake in Muscles but not in Brain

There are at least four MCT subtypes in the brain (MCT1, MCT2, MCT4, and MCT8), among which MCT4 is also a highly relevant target in glioblastoma.^18^ In the MCT4-blocked group (n = 4) in an otherwise similar experimental setting as MCT1/2 blocking, PET imaging revealed focal tracer uptake in the tumor regions, enabling clear tumor visualization (Figure 3E). Compared with the nonblocked cohort, [^18^F]­FNA tumor uptake was reduced by approximately 23%. However, the uptake was not statistically significant in the nonblocked group, as evidenced by the SUV_mean_ values in the tumor (0.41 ± 0.07, P = 0.209) and brain regions (0.34 ± 0.06, P = 0.321) and in the SUV_max_ values in tumor (0.64 ± 0.10, P = 0.156) and brain (0.53 ± 0.05, P = 0.227). The peak TBR was therefore 1.22 ± 0.37 at 5–10 min. The TACs of the brain and tumor in the MCT4-blocked group (Figure 4E) showed kinetics similar to those of the nonblocked group (Figure 4A). However, ex vivo tissue radioactivity quantification revealed statistically lower muscle uptake in the MCT4-blocked group than in the nonblocked group (Figure 6; Supplemental Table S1).

### 3.5 [^18^F]­FB PET did not Delineate the Brain Tumor

In the in silico docking analysis (Figure 8C), the nitrogen atom of FNA was found to be in an ideal hydrogen-bonding distance with the side-chain oxygen atom of S371 of MCT1, and this prompted us to study the importance of the nitrogen atom in the chemical structure of FNA for MCT1 transport. Accordingly, we prepared [^18^F]­FB (Figure 1), which does not contain a nitrogen atom but has an otherwise similar chemical structure as [^18^F]­FNA. PET imaging with [^18^F]­FB showed no focal uptake of the tracer in the tumor and negligible uptake in the brain compared with [^18^F]­FNA (Figure 3E). Brain SUV_mean_ and SUV_max_ were 0.19 ± 0.04 and 0.15 ± 0.09, respectively, both of which were significantly lower than those of [^18^F]­FNA. The lower tumor uptake was also evident in the tissue autoradiography (Supplemental Figure S3C), and no focal radioactivity uptake was observed in the corresponding tumor area.

### 3.6 In Vivo Stability and Blood Radioactivity Analysis

To facilitate the interpretation of PET imaging results, we performed tracer in vivo stability evaluation in the brain tumor mouse model. Blood samples were collected 60 min after the administration of either [^18^F]­FET or [^18^F]­FB. Blood cells, plasma proteins, and plasma supernatant were separated and radioactivity was quantified. The supernatant fraction was used for HPLC and compared with the tracer standard. Blood metabolite measurements showed that [^18^F]­FET was 90.0 ± 4.0% intact. In contrast, [^18^F]­FB showed varying extents of instability in mice (Supplemetal Figure S4). For [^18^F]­FET, blood cell binding was 45.8 ± 0.9% (n = 4), and the supernatant fraction was 50.2 ± 0.6% (n = 4), which agrees with data reported elsewhere.^19^ The corresponding plasma protein binding was 3.9 ± 0.6% (n = 4). For [^18^F]­FB, blood cell binding was 19.9 ± 1.9% (n = 3), plasma protein binding was 5.9 ± 1.5% (n = 3), and the supernatant fraction was 74.3 ± 1.5% (n = 3).

## 4 Discussion

In a previous study, we reported that [^18^F]­FNA PET could clearly delineate intracranial human BT12 glioblastoma xenograft in a mouse model.^11^ To evaluate the potential clinical applications, we conducted a comparison study using [^18^F]­FET as a benchmark and explored some aspects of the glioblastoma uptake mechanism of [^18^F]­FNA. We discovered that [^18^F]­FET PET was unable to delineate the BT12 glioblastoma xenograft in the mouse model, while [^18^F]­FNA PET clearly visualized and delineated the tumors in the same mice. This result was unexpected, albeit [^18^F]­FET PET negativity in gliomas has been previously reported. Clinical PET imaging of some patient cohorts showed that [^18^F]­FET uptake was not enhanced in 30% of low-grade and 5% of high-grade gliomas at primary diagnosis,^7^ and the underlying biological mechanism is not well understood. LAT1 (*L*-type amino acid transporter 1) is supposed to mediate [^18^F]­FET tumor uptake. However, [^18^F]­FET PET negativity did not correlate with reduced LAT1 expression in the brain tumor samples from patients.^7^ The negative [^18^F]­FET PET results prompted us to determine whether other amino acid tracers could be used to visualize the BT12 glioblastoma xenograft. Accordingly, we performed [^11^C]­MET PET in the same mouse model, which gave positive tumor imaging results. Increased physiological uptake of [^11^C]­MET in healthy brain areas was observed compared with [^18^F]­FNA PET. In general, [^18^F]­FET and [^11^C]­MET are supposed to share similar uptake mechanisms in brain tumors; however, we did not perform further studies to determine why the BT12 glioblastomas are [^18^F]­FET negative and [^11^C]­MET positive.

The progressive accumulation and retention of [^18^F]­FET in the tumor and brain tissues is in stark contrast to the kinetics of [^18^F]­FNA, in which the rapid early uptake of the tracer is followed by a continuous washout. This indicates that the tumor retention mechanisms of the tracers differ from each other. Based on the in vivo blocking experiments in a limited number of mice (n = 4), MCT1/2 appear to be an important transporter for [^18^F]­FNA uptake in intracranial glioblastoma.^11^ This result was confirmed in this study by performing additional in vivo MCT1/2 blocking experiments with a known inhibitor, the small organic compound AZD3965. This drug is a dual inhibitor of MCT1 and MCT2 with an inhibitory constant (K_i_) of 1.6 nM to MCT1 and has 6-fold higher selectivity for MCT1 than MCT2. Therefore, the role of MCT2 in [^18^F]­FNA uptake cannot be excluded under the current experimental conditions. The absence of a blocking effect on the muscle and liver indicates that the MCT1/2-mediated transport of [^18^F]­FNA in these organs is either minimal or masked by other transporters. Interestingly, only the brain showed a persistent decrease in tracer uptake both in time point comparison and overall average activity. This result was consistent with the experiments performed by different researchers with different batches of [^18^F]­FNA tracer, MCT1/2 inhibitor, and mouse glioblastoma xenograft. Thus, the inhibition experiments were reproducible and robust. In addition, we docked FNA to the cryo-EM structure of hMCT1 and identified multiple stabilizing interactions between FNA and hMCT1, further providing evidence that [^18^F]­FNA is a potential MCT1 ligand contributing to tracer uptake in the brain. It is a study limitation that we were unable to separate the roles of MCT1 and MCT2 in tumor uptake due to the lack of inhibitors with complete selectivity toward MCT1 or MCT2.

In silico docking analysis revealed that the nitrogen atom in FNA has additional interactions with MCT1. Thus, we prepared [^18^F]­FB, an [^18^F]­FNA analog without a nitrogen in the chemical structure, for PET study in the same mouse model. In a previous study,^20^ the brain uptake kinetics of [^18^F]­FB were measured in healthy monkeys, and [^18^F]­FB exhibited excellent in vivo stability. However, in our study, [^18^F]­FB was rapidly metabolized in mice. Brain uptake was low, and the glioblastoma xenograft were not delineable with PET. The in vivo instability and near-background tumor uptake of [^18^F]­FB hindered the interpretation of whether MCTs can transport [^18^F]­FB into the brain and brain tumors. In other words, the current data does not allow to conclude whether [^18^F]­FB can be transported by MCTs or not.

In the context of glioblastoma, MCT4 is another relevant target in metabolic reprogramming and tumor progression.^21^ The in vivo MCT4 blocking experiments showed a 23% reduction in tumor uptake compared with the nonblocked group. The tumor uptake measured in vivo as SUV_mean_ was not statistically different from the nonblocked group, which may also be explained by the inherent technical challenges in quantifying relatively small differences in tissue uptake in tissue boundary delineation in vivo. Among the ex vivo gamma counting of excised tissue samples, a statistically significant difference was observed in muscles. MCT4 has abundant expression in muscles,^22^ and our results indicate that [^18^F]­FNA uptake in muscles is specific. In general, the current data indicated that MCT1/2 instead of MCT4 has a major role in mediating [^18^F]­FNA uptake in the glioblastoma xenografts.

Nicotinic acid interacts with several targets, and the primary receptor is G-protein coupled receptor GPR109A, also known as hydroxycarboxylic acid receptor 2 (HCA2).^23^ GPR109A is expressed in brain,^24^ and has roles such as being a target in β-hyroxybutyrate mediated tumor cell proliferation in glioblastoma.^25^ To investigate whether GPR109A has influence on tumor uptake of [^18^F]­FNA, the radiolabeled analog of nicotinic acid, we have tested the preadministration of GPR109A inhibitor mepenzolate bromide in PET imaging of mice bearing intracranial BT12 glioblastoma xenografts. The results showed that brain tumor uptake was enhanced and tumor retention was prolonged.^11^ However, further studies are warranted to clarify whether the observed changes are due to the involvement of GPR109A or some other biological and pharmacological mechanisms.

## 5 Conclusion

Intracranial BT12 glioblastoma xenograft were negative in [^18^F]­FET PET but positive in [^18^F]­FNA and [^11^C]­MET PET. MCT1/2 and MCT4 showed important roles in [^18^F]­FNA uptake in intracranial glioblastoma xenograft and other tissues. MCT1/2 blocking significantly reduced brain and glioblastoma uptake across several cohorts studied in this and previous work. [^18^F]­FNA holds potential applications in clinical glioma PET imaging based on a completely different biological mechanism compared to current radiotracers in clinical use. Meanwhile, we must point out that the current results are based on the study in a single mouse model, and similar investigations are needed using other models with patient-derived glioma cells. Furthermore, the finding of [^18^F]­FET PET negativity in this mouse model of patient-derived glioblastoma will be valuable for studying the underlying mechanisms of glioma [^18^F]­FET negativity, as observed in 10–30% of patients with glioma, which may advance the improved use of [^18^F]­FET in clinical glioma imaging.

## Supplementary Material



## Notes

Graphic animal study design, [^18^F]­FB shelf life HPLC analysis, autoradiography and H&E staining of brain tissue sections, and tissue biodistribution tables (PDF)

## Abbreviations

FNA: 6-fluoronicotinic acid
HPLC: high-performance liquid chromatography
MCT1: monocarboxylate transporter 1
PET: positron emission tomography
SUV: standardized uptake value
TAC: time–activity curve
%ID/g: percentage of injected dose per gram (of tissue)
