# Supplementary material for: Intracranial Human BT12 Glioblastoma Xenograft is [18F]FET PET Negative but 6‑[18F]Fluoronicotinic Acid PET Positive: Exploring a Novel Approach for Clinical Glioblastoma Imaging
Source: Mol Pharm. 2026 Jun 9;23(7):3843–54. doi: 10.1021/acs.molpharmaceut.6c00279 (PMC13343506; doi:10.1021/acs.molpharmaceut.6c00279)
Supplement: Supplementary file 1 [file mp6c00279_si_001.pdf]

## Supporting Information

# Intracranial Human BT12 Glioblastoma Xenograft is [$^{18}$F]FET PET Negative but 6-[$^{18}$F]Fluoronicotinic Acid PET Positive: Exploring a Novel Approach for Clinical Glioblastoma Imaging

David Ekwe[1], Abiodun Ayo[2,3,#], Xiaoqing Zhuang[1,4,#], Pyry Dillemuth[1,4], Tomi T. Airenne[5], Emel Bakay[1,6], Petter Lövdahl[1,6], Jonne Kunnas[1,6], Lu Bai,[1] Johan Rajander[1,7], Tiina A. Salminen[5], Jessica M. Rosenholm[6], Pirjo Laakkonen[2,3,8], and Xiang-Guo Li[1,4,9,10]

*[1]Turku PET Centre, University of Turku, Turku, Finland; [2]Translational Cancer Medicine Research Program, Faculty of Medicine, University of Helsinki, Helsinki, Finland; [3]Laboratory Animal Centre, HiLIFE University of Helsinki, Helsinki, Finland; [4]Department of Chemistry, University of Turku, Turku, Finland; [5]Structural Bioinformatics Laboratory and InFLAMES Research Flagship, Biochemistry, Faculty of Science and Engineering, Åbo Akademi University, Turku, Finland; [6]Pharmaceutical Sciences Laboratory, Department of Natural and Health Sciences, Faculty of Science and Engineering, Åbo Akademi University, Turku, Finland; [7]Accelerator Laboratory, Åbo Akademi University, Turku, Finland; [8]iCAN Flagship, University of Helsinki, Helsinki, Finland; [9]Turku PET Centre, Turku University Hospital, Turku, Finland; [10]InFLAMES Research Flagship, University of Turku, Turku, Finland.*

**Correspondence:** Associate Professor Xiang-Guo Li, Turku PET Centre, University of Turku, Kiinamyllynkatu 4-8, FI-20520 Turku, Finland. ORCID 0000-0002-9118-7223. Phone: +358 50 4485069; E-mail: xiali@utu.fi

[#]Equal contribution.

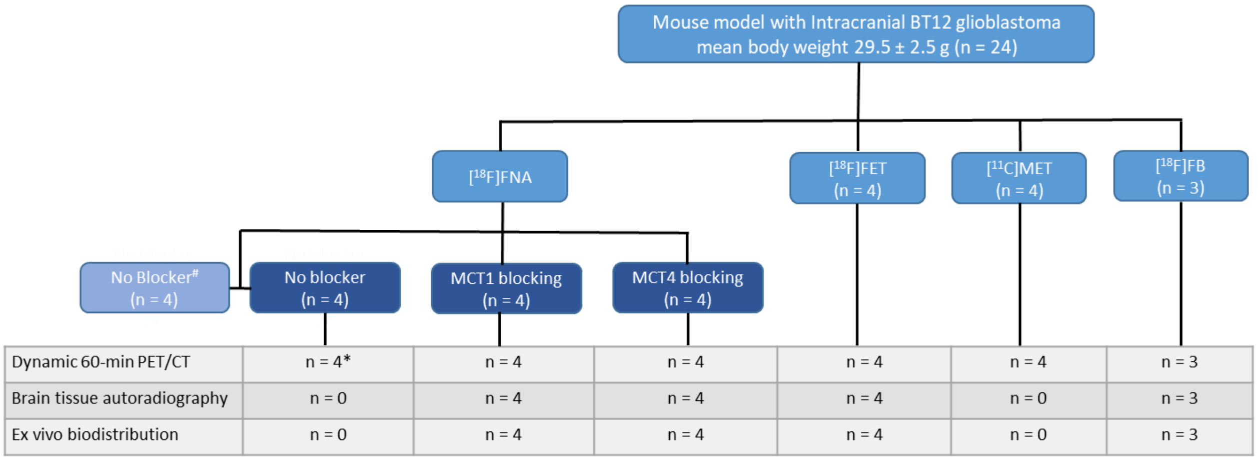


| | | | | | | |
|---|---|---|---|---|---|---|
| Dynamic 60-min PET/CT | n = 4* | n = 4 | n = 4 | n = 4 | n = 4 | n = 3 |
| Brain tissue autoradiography | n = 0 | n = 4 | n = 4 | n = 4 | n = 0 | n = 3 |
| Ex vivo biodistribution | n = 0 | n = 4 | n = 4 | n = 4 | n = 0 | n = 3 |

#Mice were only used in the [$^{18}$F]FNA ex vivo biodistribution study.
*Three mice were PET/CT imaged twice, first with [$^{18}$F]FNA and then with [$^{18}$F]FET on consecutive days; one of the mice was only PET/CT imaged with [$^{18}$F]FNA.

**SUPPLEMENTAL FIGURE S1.** Animal study design.

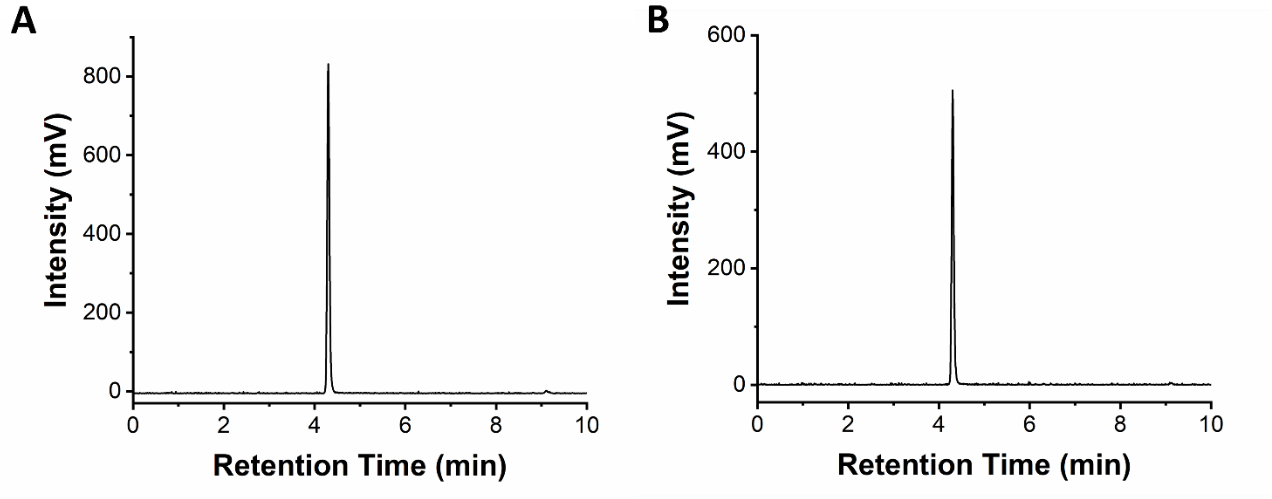


**SUPPLEMENTAL FIGURE S2.** Analytical HPLC chromatograms of [$^{18}$F]FB. (A) Radiochemical purity measurements using analytical HPLC with radioactivity detection at the end of synthesis. (B) Radiochemical purity measurements using analytical HPLC with radioactivity detection at 6 h after synthesis.

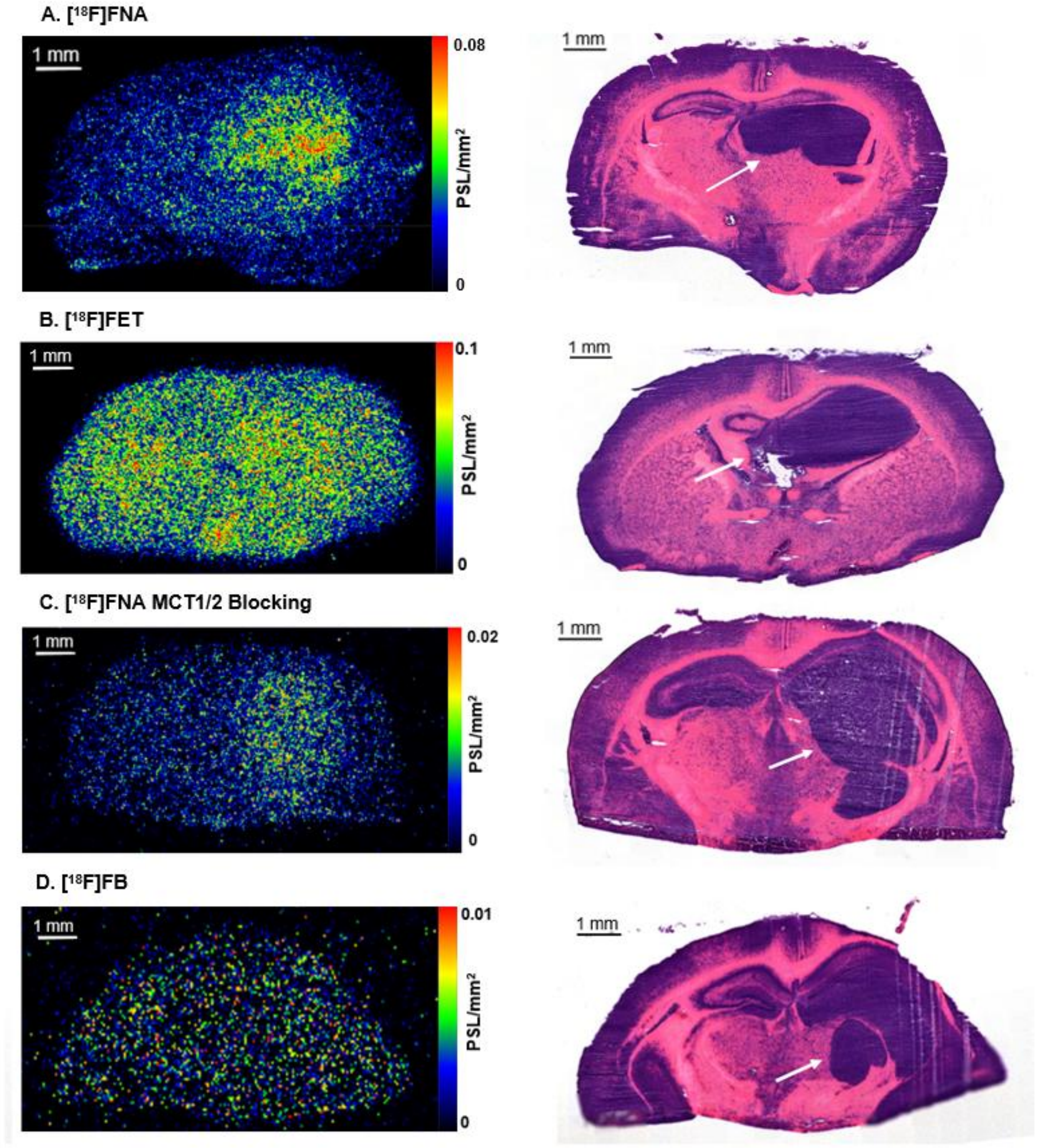


**SUPPLEMENTAL FIGURE S3.** Autoradiography and H&E images of mouse brain tissue samples with glioblastoma. Tumors were indicated with white arrows.

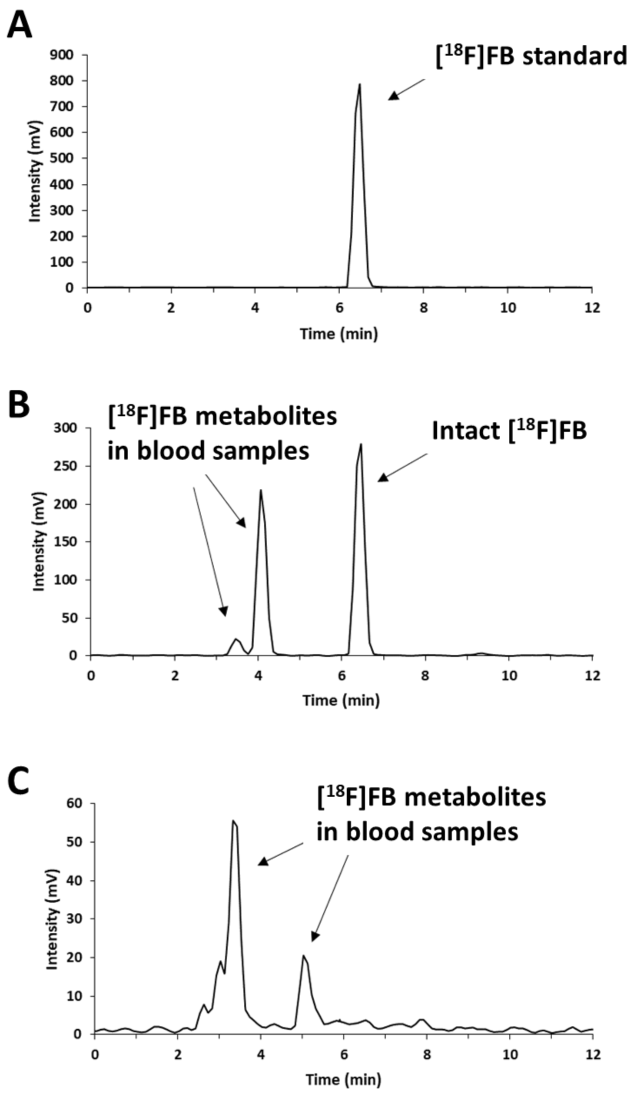


**SUPPLEMENTAL FIGURE S4.** Representative HPLC chromatograms of in vivo stability analysis of [$^{18}$F]FB in blood samples taken at 60 min post-injection from mice, showing varying extents of instability. (A) [$^{18}$F]FB standard. (B) Significant amount (approximately 50%) of intact [$^{18}$F]FB was observed in some of the blood samples, while no any intact [$^{18}$F]FB was observed some other blood samples.

**SUPPLEMENTAL TABLE S1.** Ex vivo tissue biodistribution in mice with intracranial glioblastoma xenografts with or without MCT blocking.

| | [$^{18}$F]FNA | | | [$^{18}$F]FB (n = 3) | [$^{18}$F]FET (n = 4) |
|---|---|---|---|---|---|
| | **Non-blocked** (n = 4) | **MCT1/2 blocked** (1.1 mg/kg) (n = 4) | **MCT4 blocked** (1.1 mg/kg) (n = 4) | | |
| Blood | 1.44 ± 0.91 | 0.56 ± 0.42 | 0.32 ± 0.08 | 0.13 ± 0.19 | 2.74 ± 0.21 |
| Blood cells | 0.75 ± 0.46 | 0.30 ± 0.23 | 0.17 ± 0.05 | 0.05 ± 0.08 | 2.59 ± 0.27 ***↑ |
| Bone (skull) | 1.71 ± 1.22 | 1.41 ± 0.57 | 1.15 ± 0.40 | 0.09 ± 0.07 | 1.07 ± 0.22 |
| Brain | 0.76 ± 0.42 | 0.16 ± 0.13 *↓ | 0.15 ± 0.05 | 0.03 ± 0.03 *↓ | 1.92 ± 0.17 **↑ |
| Brown fat | 0.42 ± 0.28 | 0.14 ± 0.12 | 0.16 ± 0.10 | 0.04 ± 0.03 | 1.05 ± 0.18 **↑ |
| Femur (bone + marrow) | 1.32 ± 0.96 | 1.34 ± 0.47 | 0.89 ± 0.22 | 0.10 ± 0.03 | 1.32 ± 0.35 |
| Harderian glands | 0.86 ± 0.49 | 0.38 ± 0.20 | 0.33 ± 0.08 | 0.13 ± 0.06 | 1.43 ± 0.34 |
| Heart | 0.66 ± 0.35 | 0.25 ± 0.19 | 0.20 ± 0.07 | 0.04 ± 0.04 *↓ | 1.35 ± 0.23 *↑ |
| Kidneys | 20.60 ± 22.66 | 4.81 ± 5.04 | 2.32 ± 1.06 | 0.92 ± 1.29 | 1.70 ± 0.82 |
| Liver | 0.92 ± 0.54 | 0.35 ± 0.23 | 0.25 ± 0.08 | 0.14 ± 0.17 | 1.74 ± 0.18 *↑ |
| Lungs | 0.36 ± 0.23 | 0.18 ± 0.18 | 0.12 ± 0.06 | 0.09 ± 0.07 | 1.61 ± 0.20 ***↑ |
| Muscle | 0.56 ± 0.31 | 0.19 ± 0.13 | 0.16 ± 0.09 *↓ | 0.09 ± 0.09 | 2.36 ± 0.48 ***↑ |
| Ovaries | 1.02 ± 0.65 | 0.25 ± 0.15 | 0.68 ± 0.82 | 0.34 ± 0.48 | 2.82 ± 0.67 **↑ |
| Pancreas | 0.57 ± 0.35 | 0.21 ± 0.14 | 0.15 ± 0.05 | 0.03 ± 0.03 | 21.34 ± 3.05 ***↑ |
| Plasma | 2.13 ± 1.34 | 0.79 ± 0.59 | 0.46 ± 0.11 | 0.19 ± 0.29 | 2.88 ± 0.18 |
| Spleen | 0.64 ± 0.40 | 0.25 ± 0.17 | 0.12 ± 0.09 | 0.04 ± 0.03 | 2.38 ± 0.19 ***↑ |
| Uterus | 1.47 ± 0.96 | 0.42 ± 0.15 | 0.31 ± 0.08 | 0.45 ± 0.71 | 2.63 ± 0.28 |

All mice have glioblastoma.
P values compared with the non-blocked group: *$P < 0.05$, **$P < 0.01$, and ***$P < 0.001$.

**SUPPLEMENTAL TABLE S2.** Mean values and statistics of tissue SUV at different time points in mice with intracranial glioblastoma xenografts.

| Time Point (min) | Whole Brain $SUV_{mean}$ | | | Lungs $SUV_{mean}$ | | | Muscle $SUV_{mean}$ | | | Liver $SUV_{mean}$ | | | Kidneys $SUV_{mean}$ | | |
|---|---|---|---|---|---|---|---|---|---|---|---|---|---|---|---|
| | [18F]FNA# | MCT1/2## | **P value&** | [18F]FNA | MCT1/2 | **P value** | [18F]FNA | MCT1/2 | **P value** | [18F]FNA | MCT1/2 | **P value** | [18F]FNA | MCT1/2 | **P value** |
| 5–10 | 0.65 | 0.19 | 0.001 | 0.72 | 0.57 | 0.032 | 0.53 | 0.09 | 0.61 | 1.13 | 1.00 | 0.383 | 10.35 | 6.67 | 0.015 |
| 10–15 | 0.65 | 0.18 | <0.001 | 0.63 | 0.48 | 0.009 | 0.49 | 0.12 | 0.651 | 0.94 | 0.82 | 0.29 | 8.39 | 5.53 | 0.007 |
| 15–20 | 0.60 | 0.17 | 0.0013 | 0.54 | 0.38 | 0.015 | 0.47 | 0.14 | 0.652 | 0.80 | 0.62 | 0.117 | 7.01 | 4.23 | 0.01 |
| 20–25 | 0.57 | 0.16 | 0.003 | 0.47 | 0.34 | 0.085 | 0.42 | 0.14 | 0.647 | 0.71 | 0.56 | 0.213 | 6.30 | 3.74 | 0.015 |
| 25–30 | 0.50 | 0.15 | 0.005 | 0.42 | 0.27 | 0.055 | 0.39 | 0.13 | 0.678 | 0.61 | 0.43 | 0.122 | 5.35 | 2.87 | 0.018 |
| 30–40 | 0.44 | 0.13 | <0.001 | 0.37 | 0.22 | 0.044 | 0.36 | 0.13 | 0.959 | 0.54 | 0.35 | 0.083 | 4.64 | 2.35 | 0.019 |
| 40–50 | 0.36 | 0.11 | <0.001 | 0.29 | 0.16 | 0.024 | 0.34 | 0.10 | 0.603 | 0.42 | 0.23 | 0.053 | 3.57 | 1.55 | 0.009 |
| 50–60 | 0.29 | 0.10 | 0.001 | 0.24 | 0.11 | 0.016 | 0.31 | 0.07 | 0.865 | 0.33 | 0.17 | 0.05 | 2.73 | 1.08 | 0.008 |

# Mice (n = 4) PET/CT imaged with [18F]FNA.
## Mice (n = 4) PET/CT imaged with [18F]FNA in the presence of the dual MCT1/2 blocker AZD3965 (1.1 mg/kg).
& P values < 0.05 were considered statistically significant; P values > 0.05 were not considered significant and are highlighted in red.

## PET/CT Image Analysis

PET data were reconstructed with an ordered subset expectation maximization 3-dimensional algorithm (OSEM-3D) into 6 × 10 s, 4 × 60 s, 5 × 300 s, and 3 × 600 s time frames, and CT was reconstructed using iterative image space reconstruction algorithm (ISRA). PET/CT images were analyzed using our in-house–developed Carimas 2.10 software (Turku PET Centre, Finland, www.turkupetcentre.fi/carimas/) as the major analysis tool. PET/CT images were automatically co-registered, with alignment subsequently confirmed by visual assessment of anatomical landmarks. To quantify tracer uptake in the healthy brain and tumor, regions of interest (ROIs) of identical size and volume were drawn on PET/CT brain images over the area of maximal focal uptake corresponding to the tumor, and over the contralateral hemisphere representing the normal brain tissue. The cranial injection site (needle burr hole), visible on coronal CT images was used as anatomical reference for tumor localization. In drawing the tumor ROIs, tumor boundaries were approximated based on focal areas of higher activity directly beneath this landmark. In cases where tumor uptake were not visually discernable, ROIs of same dimension were drawn at the corresponding anatomical location to estimate the tumor regions. Tumor presence and location in all the animals were confirmed by histological analysis of their postmortem hematoxylin & eosin (H&E)-stained brain sections. Additional ROIs were drawn in the right lungs, gluteal muscle, liver, kidneys caudal vena cava (blood) and whole brain. The values obtained from PET image ROIs were converted to standardized uptake values (SUVs), correcting for differences in body weight and injected radioactivity dose. The average radioactivity of a measured region over the 60-minutes PET imaging duration is reported as $SUV_{mean}$, and the highest SUV observed at a timepoint beyond 5

minutes post-injection (to exclude early phase noise) is reported as $SUV_{max}$. The tumor-to-brain ratio (TBR) is given by Tumor-$SUV_{max}$/Brain-$SUV_{max}$. Representative PET/CT brain images are time-weighted mean reconstructions of frames within the indicated time intervals, with the time window selected for each subject and tracer to display the highest tumor-to-brain contrast as determined by the time-activity curves.
